# *De Novo* Assembly and Characterization of Early Embryonic Transcriptome of the Horseshoe Crab *Tachypleus tridentatus*

**DOI:** 10.1371/journal.pone.0145825

**Published:** 2016-01-05

**Authors:** Mingliang Chen, Chenying Wang, Wei Wang, Gubiao Ji, Bin Hu, Mi Du, Guosheng Liu, Zengpeng Li, Weiyi Wang, Xiangzhi Lin, Weibing Zheng, Jianming Chen

**Affiliations:** 1 State Key Laboratory Breeding Base of Marine Genetic Resources, South China Sea Bio-Resource Exploitation and Utilization Collaborative Innovation Center, Third Institute of Oceanography, State Oceanic Administration, Xiamen, Fujian Province, China; 2 Center of Marine Biotechnology, Third Institute of Oceanography, State Oceanic Administration, Xiamen, Fujian Province, China; University of Maryland, College Park, UNITED STATES

## Abstract

The horseshoe crab *Tachypleus tridentatus* is a unique marine species and a potential model for marine invertebrate. Limited genomic and transcriptional data are currently available to understand the molecular mechanisms underlying the embryonic development of *T*. *tridentatus*. Here, we reported for the first time the *de novo* transcriptome assembly for *T*. *tridentatus* at embryonic developmental stage using Illumina RNA-seq platform. Approximate 38 million reads were obtained and further assembled into 133,212 unigenes. Sequence homology analysis against public databases revealed that 33,796 unigenes could be annotated with gene descriptions. Of the annotated unigenes, we identified a number of key components of several conserved metazoan signaling pathways (Hedgehog, Wnt, TGF-beta and Notch pathways) and other important regulatory genes involved in embryonic development. Targeted searching of *Pax* family genes which play critical roles in the formation of tissue and organ during embryonic development identified a complete set of *Pax* family genes. Moreover, the full length *T*. *tridentatus Pax1/9a* (*TtPax1/9a*) and *Pax1/9b* (*TtPax1/9b*) cDNA sequences were determined based on the transcriptome, demonstrating the immediate application of our database. Using quantitative real time PCR, we analyzed the expression patterns of *TtPax1/9a* and *TtPax1/9b* in different tissues of horseshoe crab. Taking advantage of Drosophila model, we further found that *TtPax1/9b*, but not *TtPax1/9a*, can partly rescue the Drosophila homolog *Poxm* dysfunction-caused lethality at the larval stage. Our study provides the embryonic transcriptome of *T*. *tridentatus* which could be immediately used for gene discovery and characterization, functional genomics studies in *T*. *tridentatus*. This transcriptome database will also facilitate the investigations of molecular mechanisms underlying embryonic development of *T*. *tridentatus* and other marine arthropods as well.

## Introduction

Emergence of new model organisms plays increasingly critical roles in embryogenesis and evolutionary developmental study. Until now, the most well-known model organism in arthropods is the fruit fly *Drosophila melanogaster* (belonging to Class Insecta), which is widely used in studies of genetics and embryogenesis [1,2]. The genome of water flea *Daphnia pulex* (belonging to Class Crustacea) was also published recently, which facilitates the study of cellular response to environmental challenges [3]. Nevertheless, due to lack of genomic information, understanding of developmental and molecular mechanisms of most arthropods is still lagged far behind that of the vertebrates, which consequentially hinders the appearance of new model organisms within arthropods. Fortunately, high throughput *de novo* transcriptome assembly has proven to be a valuable technology to obtain sequence information and expression level of large-scale target genes involved in a particular biological process without any prior knowledge of reference genome [4–7]. In fact, this technology has been applied to analyze transcriptomes from a variety of species in metazoan [8–12].

Horseshoe crabs (Family Limulidae, Order Xiphosura, Class Merostomata), which are an extremely ancient marine group, have emerged as a valuable laboratory animal model in developmental study of marine invertebrate for decades [13–16]. They inhabit the areas around the shallow coastal seas and breed on intertidal shores. Even though horseshoe crabs show some common features of crustaceans (crab-like shell and claws), they are more closely related to arachnids, such as spiders and scorpions [17,18]. To date, only four extant horseshoe crab species have been discovered in two distinctly separate regions of the world, viz. *Tachypleus tridentatus*, *Tachypleus gigas*, *Carcinoscorpius rotundicauda* (mainly found in Southeast Asia) and *Limulus polyphemus* (only found in western Atlantic coast of North America). *T*. *tridentatus* was once very common along the southeast coast of Mainland China. Unfortunately, its population has been declining for several decades due to excessive hunting by humans and environmental pollution. Fossil evidence revealed that the earliest horseshoe crab lived during the late Ordovician period, around 445 million years ago [19]. More strikingly, the horseshoe crab remains unchanged in overall morphology for over 200 million years, and therefore is considered as a “living fossil” [20]. Besides their importance for the evolutionary studies and preservation of ecological diversity, horseshoe crabs also serve as a multiple-use animal resource. For example, the Limulus Amebocyte Lysate test, which is widely applied in the detection and quantification of bacterial endotoxins, is based on an aqueous extract of amebocytes from horseshoe crab [21]. Moreover, it is believed that horseshoe crabs are essential for the maintenance of the ecology of estuarine and coastal communities [22].

It is reported that the horseshoe crabs take about 10–15 years to reach sexual maturity from fertilized eggs and more than ten molts occur during this period [23], which makes it difficult to record the whole growth process of horseshoe crab in natural habitat without interruption. Growth observation of horseshoe crabs from larvae to adulthood in laboratory also turned out to be a failure [24]. On the other hand, over the recent decades, the horseshoe crab has emerged as an experimental model for studying marine invertebrate embryology, structure-function relationship of the visual system and nervous system [15,25]. Morphological changes during early embryonic development of two horseshoe crab species, including *T*. *tridentatus* and *L*. *polyphemus*, have been studied [14,23]. According to Sekiguchi’s classification [23], the embryonic development of horseshoe crab could be roughly divided into the 21 stages, mainly including cleavage, blastula, gastrula, appearance of germ disc, formation of appendages and finally hatch-out to the first instar “trilobite larvae”. During hatch-out stage, the embryo grows remarkably and several organs further develop. For instance, the central eye becomes discernable as a brownish spot. The appendages of the prosoma are further expanded [23]. All these changes enable the horseshoe crab to survive in the new challenging circumstance without the protection of chorion.

Although the study of horseshoe crab morphological changes during embryonic development has been described, the detailed molecular mechanism underlying this process remains unknown, which is largely due to the lack of genomic information. In the present study, we employed high-throughput Illumina Solexa sequencing and gene annotation to characterize the transcriptome of *T*. *tridentatus* embryo at the hatch-out stage. We reported for the first time a comprehensive analysis of large-scale gene expression profile during *T*. *tridentatus* embryonic development, which could be immediately used for further gene discovery and functional genomics study. Our data indicated that the major signaling pathways and key regulatory factors involved in embryonic development were highly conserved between *T*. *tridentatus* and other metazoans, especially *D*. *melanogaster*. Molecular cloning and functional study of *T*. *tridentatus Pax1/9a* and *Pax1/9b* were also investigated in our study. Therefore, the transcriptome analysis reported here have important applications to the understanding of molecular mechanisms underlying *T*. *tridentatus* embryonic development.

## Materials and Methods

### Horseshoe crab maintenance and breeding

Adult *T*. *tridentatus* were maintained in a 3 m×1 m×2 m tank containing natural seawater (temperature 25°C, salinity 30 ppt) and fed with bivalves. Fertilized eggs were obtained by natural spawning and cultured in the laboratory with standard procedures [26]. The staging of embryos was according to Sekiguchi’s developmental tables [23]. The embryos at Stage 21 (the hatch-out stage) were collected for high throughput transcriptome sequencing.

### RNA extraction and quality determination

Total RNA of *T*. *tridentatus* embryos was isolated by TRIzol (Invitrogen, Carlsbad, CA, USA) according to the standard protocol. The RNA samples were treated with DNase I (TaKaRa, Japan) for 4 h. RNA was quantified by measuring the absorbance at 260 nm using a NanoDrop spectrophotometer (Thermo Fisher Scientific Inc., San Jose, CA, USA). The purity of RNA was assessed by the ratio of the absorbance at 260 and 280 nm. The integrity of the RNA samples was examined with an Agilent 2100 Bioanalyzer (Agilent Technologies, Santa Clara, CA, USA).

### Cloning of full length *T*. *tridentatus Pax1/9* cDNAs

To obtain the full length cDNAs of *Pax1/9* genes, the BLAST search of human *Pax1* and *Pax9* genes were performed against the *T*. *tridentatus* transcriptome database, which resulted in two sequences with high homology. The 3’and 5’ ends were obtained by rapid amplification of cDNA ends (RACE) approaches using 3’-Full RACE Core Set with PrimeScript™ RTase and 5’-Full RACE Kit with TAP (TaKaRa, Japan) following the manufacturer’s instructions. Primers for 3’-RACE and 5’-RACE were listed in S1 Table. The PCR products were ligated into pMD-19T vector (TaKaARa, Japan) and transformed into the competent *E*. *coli* TOP10 cells. Positive clones with the expected-size inserts were determined by colony PCR and DNA sequencing.

### cDNA library preparation, Illumina sequencing and sequence assembly

The cDNA library was prepared using the TruSeq^TM^ RNA Sample Preparation Kit (Illumina, San Diego, CA, USA) according to the manufacturer’s instructions. Poly(A)-containing mRNA was purified by Oligo(dT) magnetic beads from 10 μg total RNA sample and fragmented using divalent cations. The cleaved RNA fragments were used for the first strand cDNA synthesis using reverse transcriptase and random primers, followed by second strand cDNA synthesis using DNA polymerase I and RNase H. After second strain cDNA synthesis, fragments were treated with end repair, A-base tailing and adapter ligation consecutively. The sample was further treated by gel size fractionation and PCR amplification to create final cDNA library. The cDNA library was sequenced on the Illumina Cluster Station and Illumina Genome Analyzer system according to the manufacturer’s instructions. The Trinity method was used for *de novo* assembly of Illumina reads of *T*. *tridentatus* embryos [27]. Briefly, the trinity using *de Bruijn* graph algorithm was run on the paired-end sequences with the fixed default *k*-mer size of 25, minimum contig length of 200 and paired fragment length of 500.

### Functional annotation

All possible coding sequences were predicted by GetORF model of EMBOSS (http://emboss.sourceforge.net/apps/cvs/emboss/apps/getorf.html) with default parameters. The longest ORF was considered as the candidate coding sequence (CDS). The assembled unigenes were annotated based on sequence similarity by sequential BLAST searches against National Center for Biotechnology Information (NCBI) non-redundant protein database (NR) and nucleotide sequences database (NT), the Swiss-Prot protein database, the Kyoto Encyclopedia of Genes and Genomes (KEGG) pathway database, the Cluster of Orthologous Groups (COG) database and the Translated EMBL Nucleotide Sequence Database (TrEMBL). The Blast2GO software was used for blasting and assigning associated gene ontology (GO) terms describing biological processes, molecular functions and cellular components.

### Phylogenetic analysis

Related sequences from *homo sapiens* and *Drosophila melanogaster* were included in the phylogenetic analysis (S2 Table). The DNA binding domains of Pax family proteins was aligned manually and Mega 3 [28] was used to generate the phylogenetic trees. The neighbor joining method with 1000 bootstrap replications was used to calculate each tree.

### Expression of TtPax1/9a and TtPax1/9b in T. tridentatus tissues

To assess the tissue distribution of *TtPax1/9a* and *TtPax1/9b* transcripts, total RNA was extracted from intestine, liver, yellow connective tissue, heart, stomach, muscle and gill, respectively. First strand cDNA synthesized from 2 μg total RNA was used as templates for quantitative real time PCR using IQ^TM^ 5 Multicolor Real-time PCR Detection System (Bio-Rad, Richmond, CA, USA). The TtPax1/9a specific primers are Pax1/9a-Fw (5’-AGCCGTTTACCTGAATCGAC-3’) and Pax1/9a-Rv (5’-AAATATTGTGCACTTGCTGGA-3’). The TtPax1/9b specific primers are Pax1/9b-Fw (5’-CCAGTGTCCATGCCATTAAG-3’) and Pax1/9b-Rv (5’-GTTTGCGGTGACACTGTTCT-3’). The *T*. *tridentatus* GAPDH gene was used as the internal control. Specific primers for *T*. *tridentatus* GAPDH are GAPDH-Fw (5’-ATCATCAGCAATGCCTCTTG-3’) and GAPDH-Rv (5’-GCCTTAGAGCTTGGTCCATC-3’).

### Generation of transgenic flies

*D*. *melanogaster* were cultured on standard medium with 12/12 light/dark cycle at 25°C. The *poxm* loss-of-function mutant fly *Poxm*^*R361*^ and transgenic line *poxm8*.*4-Gal4* have been described previously [29]. Taking advantage of *EcoR* I and *Xho* I restriction sites, the full length cDNA of *TtPax1/9a* and *TtPax1/9b* were subclone into pUAST vector. The *UAS-TtPax1/9a* and *UAS-TtPax1/9b* transgenic fly lines were generated by germline transformation with co-injection of the helper plasmid Δ2–3 according to standard protocol [30]. The *UAS-TtPax1/9a* or *UAS-TtPax1/9b* transgenic fly lines were crossed with *poxm8*.*4-Gal4* fly to obtain the *TtPax1/9a*- and *UAS-TtPax1/9b*-expressing flies under the control of *poxm8*.*4* upstream region. The transgenic flies were imaged with Leica M205FA microscope (Leica, Wetzlar, German).

## Results and Discussion

### Sequencing and transcriptome assembly

In this study, 10 μg of total RNA isolated from the *T*. *tridentatus* embryos at hatch-out stage were used to prepare cDNA library for subsequent transcriptome sequencing using an Illumina HiSeq2000 sequencer. RNA was pooled from multiple *T*. *tridentatus* embryos to prepare one sample for sequencing. The summary of Illumima sequencing and annotation was shown in Table 1. Sequencing of cDNA library generated a total of 37,902,792 paired end reads. These data are available from the NCBI Short Read Archive under accession number SRR946952. Transcriptome assembly was completed using RNAseq *de novo* assembler Trinity (*k*-mer length = 25). 4,864,778 contigs were identified with the nucleotide composition of A, T, C and G being 30.98%, 29.67%, 20.22% and 19.12% respectively, which gives rise to an overall GC content of 39.34% in the whole transcriptome. The contigs were further assembled into 176,231 transcripts falling into 133,212 unigenes (> 200 bp) with a mean unigene length of 746 bp and an N50 of 568 bp (Table 1). The length distribution of transcripts and unigenes were shown in S1 Fig.

**Table 1:** Summary of *T*. *tridentatus* transcriptome sequencing, assembly and annotation.

Stage		Data
Sequencing	Sequencing reads	37,902,792
Assembly	Total transcripts	176,231
	N50 length of transcripts (bp)	1147
	Mean length of transcripts (bp)	713
	Max length of transcripts (bp)	11,337
	Total unigenes	133,212
	N50 length of unigenes (bp)	746
	Mean length of unigenes (bp)	568
	Max length of unigenes (bp)	11,337
Annotation	Total unigenes annotated	33,796
	Total unigenes unannotated	99,416
	Unigenes annotated against NR database	29,918
	Unigenes annotated against NT database	11,116
	Unigenes annotated against Swiss-Prot database	24,118
	Unigenes annotated against TrEMBL database	29,884
	Unigenes annotated against GO database	15,768
	Unigenes annotated against COG database	6,920
	Unigenes annotated against KEGG database	10,999

Unigene annotation was achieved by BLASTx and BLASTn searches against NR, NT, Swissprot, and TrEMBL with an e-value less than 1×10^−5^. In total, there were 33,796 unigenes showing hits in one or more databases (Table 1), among which the NR database gave rise to the most annotations (29,918 hits). However, a large proportion of the sequences did not show significant blast hit. This was probably due to the lack of characterization of genes from closely related species, as the length distribution and the coverage of the annotated and unannotated unigenes were in a similar pattern (S2 Fig). The assembled sequences generated in our study are available from the authors upon request.

### Functional annotation

**Fig 1:**
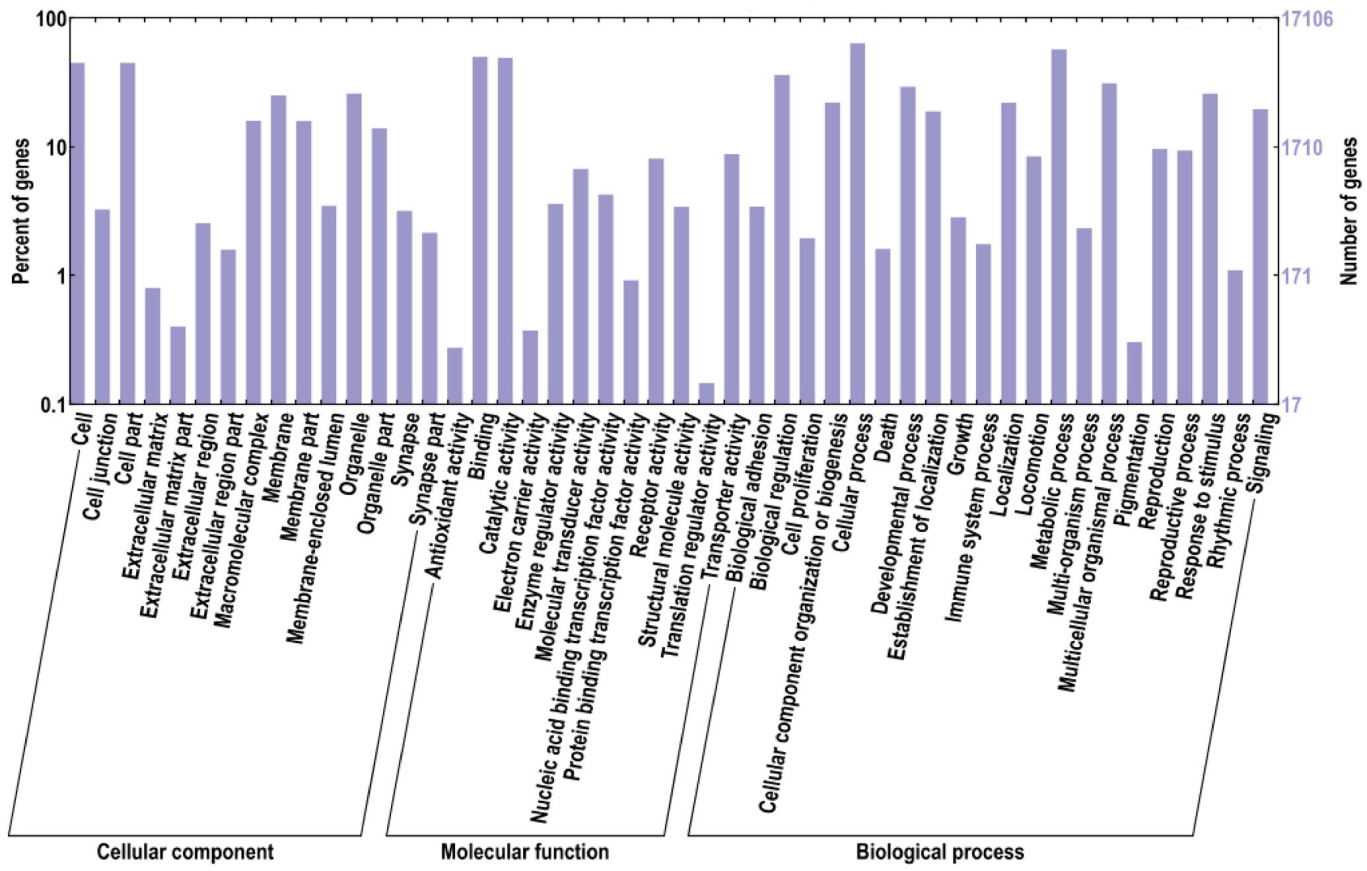
GO distribution of the T. tridentatus unigenes. Go ontology distribution of the T. tridentatus unigenes were derived using BLAST2GO. The X-axis represents three main GO categories: cellular component, molecular function and biological process, which further separated into 15, 12 and 21 functional groups, respectively. The Y-axis represents percentages and numbers of unigenes mapping to the given functional GO group.

In order to predict the functions of these unigenes, all the sequences were analyzed according to gene ontology (GO) database and clusters of orthologous groups (COGs). 15,768 genes were successfully annotated with 95,305 GO terms and were separated into three categories: biological process, cellular component, and molecular function, which were further divided into 15, 12 and 21 functional groups, respectively (Fig 1). Among these GO terms, 49,724 unigene sequences were assigned to biological process, 27,948 to molecular function, and 17,363 to cellular component. Interestingly, the top 5 groups with most unigenes involved in the biological process were: cellular process, metabolic process, biological regulation, developmental process, and multicellular organismal process. While the cell part and the cell functional groups were dominant in the cellular component category, and the binding and the catalytic activity were dominant in the molecular function category. The integral to membrane (GO: 0016021) contained 1572 unigenes, which belonged to the cellular component category. It had the largest number of unigenes among all the groups. In addition, the ATP binding (GO: 0005524) containing 1478 unigenes and the oxidation-reduction process (GO: 0055114) containing 619 unigenes were the most represented GO terms for the molecular function and biological process categories, respectively. A high percentage of unigenes involved in the following functional groups should also be noted: cellular process (GO: 0009987) with 561unigenes, protein phosphorylation (GO: 0006468) with 596 unigenes and regulation of transcription, DNA-dependent (GO: 0006355) with 556 unigenes. We were interested in the embryonic development process of *T*. *tridentatus*, and thus investigated the unigenes with GO terms containing “embryo” or “development”. As a result, a total of 183 GO terms were found with unigene numbers ranging from 1 to 241. The top 20 terms with the most number of unigenes were listed in Table 2. This demonstrated that our transcriptome database contained a variety of unigenes related to the embryonic development, which provided an abundant resource for further investigation of regulatory mechanisms of *T*. *tridentatus* embryonic development.

**Table 2:** Top 20 GO terms with most unigenes involved in embryonic development.

GO term	GO index	No. of unigenes
mushroom body development	0016319	241
peripheral nervous system development	0007422	207
mesoderm development	0007498	173
central nervous system development	0007417	162
multicellular organismal development	0007275	153
open tracheal system development	0007424	146
muscle organ development	0007517	140
embryo development ending in birth or egg hatching	0009792	125
compound eye development	0048749	113
neuromuscular junction development	0007528	105
embryonic development via the syncytial blastoderm	0001700	86
wing disc development	0035220	86
nervous system development	0007399	85
nematode larval development	0002119	82
organ development	0048513	75
chaeta development	0022416	74
gonad development	0008406	69
heart development	0007507	68
instar larval development	0002168	65
sensory organ development	0007423	62

**Fig 2:**
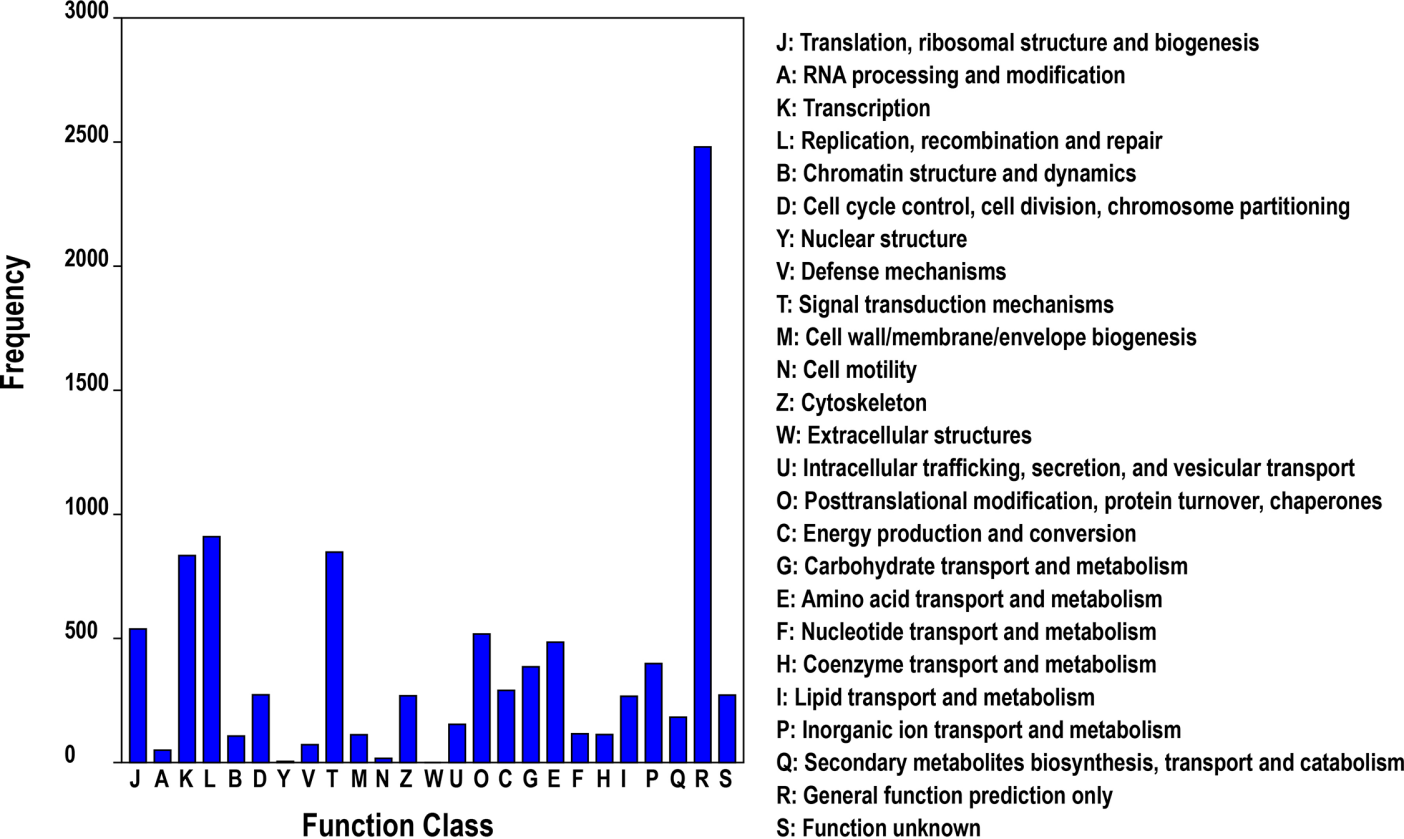
COG functional classification of the *T*. *tridentatus* transcriptome.

Meanwhile, COG annotation was used for phylogenetic classification of the putative proteins from the *T*. *tridentatus* transcriptome, in which a total of 6,920 proteins were assigned to 25 clusters (Fig 2). Among the COG library, the cluster of “General function prediction only” consisted of the largest number of unigenes. The other five largest categories were: “replication, recombination and repair” (14.47%), “signal transduction mechanisms” (13.42%), “transcription” (13.12%), “translation, ribosomal structure and biogenesis” (8.44%), and “posttranslational modification, protein turnover, chaperones” (8.24%), suggesting the specific responses and molecular mechanisms of *T*. *tridentatus* early development to some extent.

To investigate the biological pathways which are possibly involved in early embryonic development process of T. tridentatus, we further mapped the unigenes to the reference canonical pathways in the Kyoto Encyclopedia of Genes and Genomes (KEGG) database. A total of 10,999 unigenes were assigned to 298 KEGG pathways. The pathways most represented by numbers of unigene sequences were “transcription factors” (865 unigenes), “chromosome” (802 unigenes), “ubiquitin system” (798 unigenes), “protein kinases” (784 unigenes), and “pathways in cancer” (518 unigenes).

### Signaling pathways and regulatory genes involved in the development of *T*. *tridentatus*

**Fig 3:**
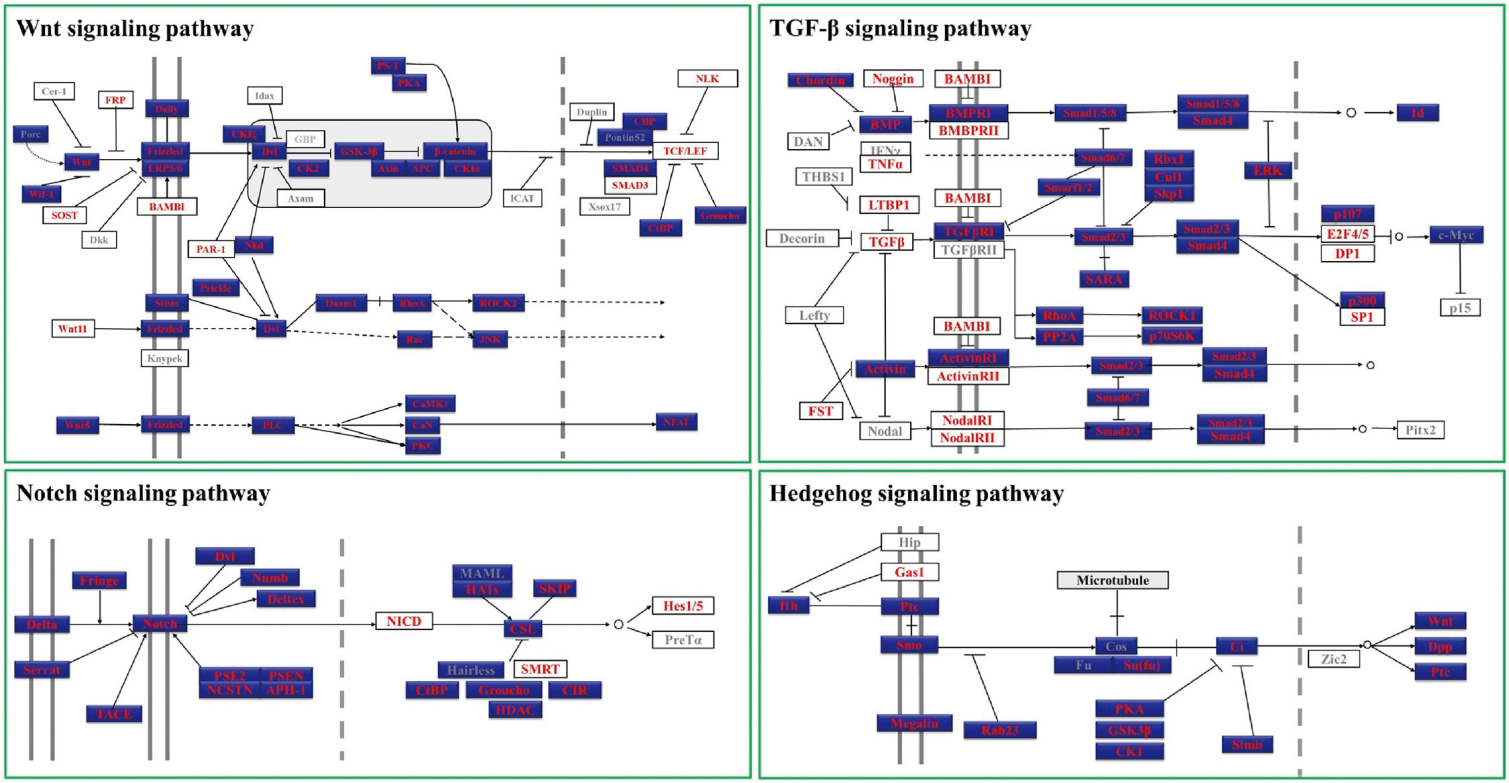
Major components from Conserved metazoan signaling pathways identified in *T*. *tridentatus*. The results were generated by BLASTing the unigenes of *T*. *tridentatus* to known homologues with the e-value cutoff at 1e-10. Pathway schematics were adapted from KEGG pathway models (http://www.genome.jp/kegg/). *Drosophila melanogaster*-specific pathways are colored blue. Genes identified and not identified in *T*. *tridentatus* were marked with red font and grey font respectively.

Formation of different cell types and patterns during development of multicellular organisms depends on a complex cascade of developmental decisions. An evolutionarily conserved network of signaling pathways and regulatory genes plays key roles during this process [31]. Analyzing these pathways and factors involved in the early embryonic development of *T*. *tridentatus* is of great importance for understanding of the developmental mechanisms of this ancient marine creature and may provide insights into the evolutional study of conserved signaling pathways and regulatory genes. In all the annotated unigenes of *T*. *tridentatus* transcriptome, we identified a number of key components of four major signaling pathways (including Hedgehog, Wnt, transforming growth factor-β and Notch pathways) conserved in metazoan. As shown in Fig 3, 98 possible homologues were identified from 126 key genes involved in these signaling pathways, representing 77.78% coverage of the total genes. The percentage of genes found in each pathway is 78.85% (Wnt), 77.27% (TGF-β), 77.78% (Hedgehog) and 87.5% (Notch) respectively. In case of *D*. *melanogaster*-specific pathways, the percentage was much higher, about 91.57% (76/83) homologues could be found in *T*. *tridentatus* transcriptome, indicating the higher similarity of *T*. *tridentatus* with *D*. *melanogaster* than other animal models in the aspect of signaling transduction pathways. It should also be noted that the sequence length of most gene fragments (> 200 bp) is sufficient for functional studies of these genes by modern molecular technology, such as real-time PCR quantification, *in situ* hybridization and antibody preparation. Moreover, with the sequence information of these gene fragments, it would be more efficient and reliable to obtain the full length of desired genes comparing with common degenerate PCR method. This is particularly helpful in case of gene discovery and functional study in the “non-model” organism horseshoe crab.

Regulatory genes are also key players in the network governing embryonic development [32]. *D*. *melanogaster* is one of the best understood models of embryonic development, especially pattern formation process, and thus we analyzed the regulatory genes potentially involved in the *T*. *tridentatus* axis specification and patterning by using *D*. *melanogaster* as a reference model. The genetic control of axis specification and patterning in *D*. *melanogaster* requires a cascade of gene regulation events before the onset of blastoderm stage. The major genes involved in this developmental process of *D*. *melanogaster* can grouped into four categories, viz. maternal effect genes, gap genes, pair rule genes and segment polarity genes [33,34]. This developmental process starts with the diffusion of the maternal effect genes which are responsible for setting the anterior-posterior polarity of the embryo. The gap genes, which are among the first genes transcribed in the embryo, participate in the establishment of the sub-domain of body plan under the control of maternal gradients. The pair rule genes subsequently divide the embryo into periodic units, whereas the segment polarity genes activated by pair rule genes further establish the periodicity of the embryo by dividing it into 14 segment-wide units [35,36]. Key regulatory genes of each category used in our study were selected according to S.F. Gilbert’s and R.M. Twyman’s classification [37,38]. Among all the 53 genes listed in Table 3, we identified a total of 40 genes (75.47%) which had homologs in *T*. *tridentatus*, suggesting that functions of these genes conserved in *T*. *tridentatus*. It should be pointed out that the percentage of gene homologs identified in the maternal effect genes category was much lower (11/18, 61.11%) comparing to that of the other categories: gap genes (12/14, 85.71%), pair rule genes (7/8, 87.5%) and segment polarity genes (13/16, 81.25%). This is possibly because the mRNA products of the maternal gene homologs have been consumed at the hatch-out stage, the last stage of *T*. *tridentatus* embryonic development. In addition, we also failed to identify some well-studied gene homologs (including *knirps*, *tailless*, *even-skipped*, *invected*, *fused* and *costal 2*), which may be due to the extremely low expression of these gene homologs in our sample. Anyway, we identified the majority of the candidate regulatory genes that are potentially involved in the axis specification and patterning of *T*. *tridentatus* embryo. Further study is required to characterize the function of these putative genes.

**Table 3:** Representative regulatory genes involved in the early embryonic development of *T*. *tridentatus*.

Name	Length (range)	No. of transcripts	Read number	Hit ID	target organism	E-value
**Major maternal effect genes**						
*bicoid*	215–717	7	84	gb|EHJ73092.1|	*Danaus plexippus*	3E-96
*hunchback*^*a*^	281	2	3	emb|CAK50841.1|	*Glomeris marginata*	1E-13
*caudal*^*a*^	286–1412	5	967	ref|XP_002405030.1|	*Ixodes scapularis*	5E-06
*staufen*	3588	1	3580	gb|ELT94341.1|	*Capitella teleta*	3E-141
*oskar*	214–4349	14	13341	ref|XP_004932537.1|	*Bombyx mori*	2E-34
*tudor*	480–1849	4	358	gb|EKC34332.1|	*Crassostrea gigas*	1E-11
*vasa*	257–3575	2	4032	gb|ACT35657.1|	*Haliotis asinina*	2E-180
*pumilio*	274–322	2	5	ref|XP_002601469.1|	*Branchiostoma floridae*	1E-45
*torso*	256–344	5	22	ref|XP_002408935.1|	*Ixodes scapularis*	2E-10
*trunk*	236–2925	13	754	gb|EFX70037.1|	*Daphnia pulex*	3E-03
*rolled*	442–858	2	58876	gb|ADV40083.1|	*Latrodectus hesperus*	6E-59
Maternal effect genes not found in *T*. *tridentatus* transcriptome: *exuperantia*, *swallow*, *nanos*, *valios*, *torsolike*, *nasrat* and *polehole*.						
**Major gap genes**						
*hunchback*^*a*^	281	2	3	emb|CAK50841.1|	*Glomeris marginata*	1E-13
*krüppel*	369–4091	3	1306	ref|XP_002435294.1|	*Ixodes scapularis*	4E-09
*giant*	468–1490	4	210	ref|XP_002429035.1|	*Pediculus humanus corporis*	5E-43
*caudal*^*a*^	286–1412	5	967	ref|XP_002405030.1|	*Ixodes scapularis*	5E-06
*huckebein*	321	1	322	ref|NP_524221.1|	*Drosophila melanogaster*	9E-27
*buttonhead*	388	1	7	ref|XP_004375586.1|	*Trichechus manatus latirostris*	5E-59
*cap'n'collar*	522–3345	4	1471	gb|EFA11557.1|	*Tribolium castaneum*	5E-46
*knot*	652–787	2	118	gb|EEZ99300.1|	*Tribolium castaneum*	7E-108
*crocodile*	289–588	2	28	emb|CAK50838.1|	*Glomeris marginata*	3E-47
*empty spiracles*	209–1313	2	142	ref|NP_001107793.1|	*Tribolium castaneum*	3E-47
*orthodenticle*	226–975	5	83	gb|AAU85255.1|	*Euscorpius flavicaudis*	2E-05
*sloppy paired*^*b*^	336	1	2	ref|NP_001071091.1|	*Tribolium castaneum*	1E-62
Gap genes not found in *T*. *tridentatus* transcriptome: *knirps* and *tailless*.						
**Major pair rule genes**						
*fushi tarazu*	240–714	3	45	ref|XP_002406407.1|	*Ixodes scapularis*	6E-17
*hairy*	242–2184	8	355	gb|EFA13687.1|	*Tribolium castaneum*	1E-29
*runt*	499–2102	3	696	emb|CAB89493.1|	*Cupiennius salei*	7E-75
*odd-paired*	1488	1	72	ref|NP_001158430.1|	*Saccoglossus kowalevskii*	7E-169
*odd-skipped*	691–2683	4	1333	ref|XP_002399401.1|	*Ixodes scapularis*	3E-79
*paired*	363	1	11	gb|ACT79975.1|	*Nasonia vitripennis*	5E-62
*sloppy paired*^*b*^	336	1	2	ref|NP_001071091.1|	*Tribolium castaneum*	1E-62
Pair rule genes not found in *T*. *tridentatus* transcriptome: *even-skipped*.						
**Major segment polarity genes**						
*engrailed*	565–1432	2	154	gb|AAB40144.1|	*Branchiostoma floridae*	3E-48
*hedgehog*	224–569	2	15	emb|CAE83646.1|	*Glomeris marginata*	3E-16
*patched*	680–3166	2	550	dbj|BAI83404.1|	*Parasteatoda tepidariorum*	5E-103
*smoothened*	1739	1	170	dbj|BAI83345.1|	*Parasteatoda tepidariorum*	3E-48
*pangolin*	473–3616	3	711	gb|EKC28444.1|	*Crassostrea gigas*	4E-75
*decapentaplegic*	390–2164	6	280	gb|AEK81570.1|	*Ptychodera flava*	3E-45
*wingless*	1711	1	135	ref|XP_002403230.1|	*Ixodes scapularis*	6E-14
*cubitus interruptus*	405–1012	3	99	ref|XP_002435743.1|	*Ixodes scapularis*	6E-43
*frizzled*	290–1569	8	292	ref|XP_002433979.1|	*Ixodes scapularis*	6E-39
*Disheveled-1*	706–1987	2	254	ref|XP_002403641.1|	*Ixodes scapularis*	1E-07
*shaggy*	526	1	15	gb|EFN78376.1|	*Harpegnathos saltator*	1E-08
*armadillo*	350–657	5	75	gb|EKC38770.1|	*Crassostrea gigas*	1E-73
*gooseberry*	771	1	23	gb|EHJ65894.1|	*Danaus plexippus*	2E-103
Segment polarity genes not found in *T*. *tridentatus* transcriptome: *invected*, *fused* and *costal 2*.						

^*a*^ gene grouped in both maternal effect genes and gap genes

^*b*^ gene grouped in both gap genes and segment polarity genes

### Case study: Detailed analysis of *Pax* family genes

**Fig 4:**
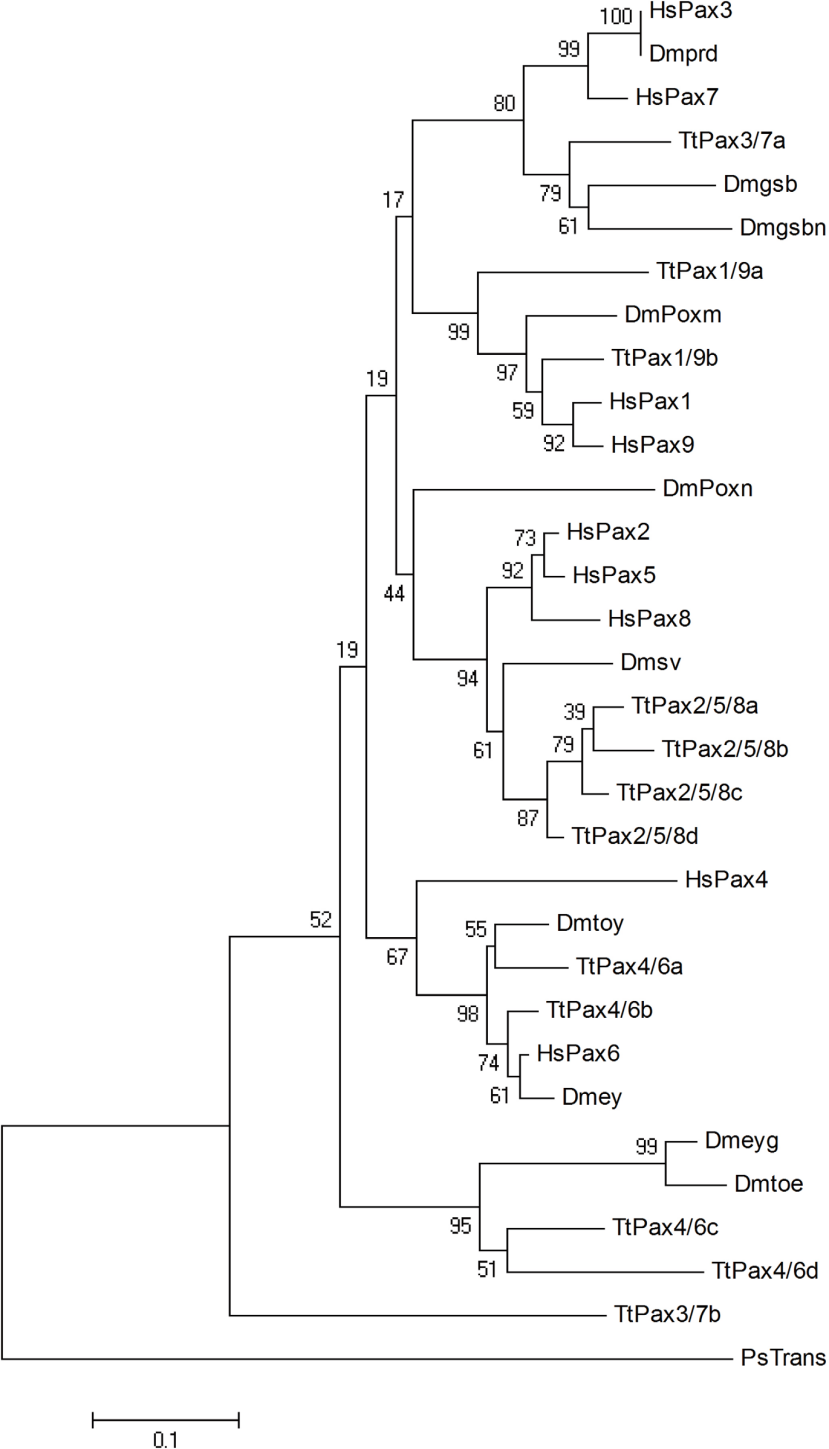
Phylogenetic analysis of paired domains of T. tridentatus Pax family proteins. Neighbour joining tree of the paired domains of Pax proteins with paired deletion option. Since TtPax4/6 has only partial paired domain available, the phylogeny of Pax4/6 subfamily was constructed with complete deletion option. A pseudomonas transposase sequence (AF169828) serves as outgroup. Numbers above branches are the percentage of the trees in which the topology appears. Paired domain sequences of Pax proteins were shown in S2 Table. Tt: *Tachypleus tridentatus*. Hs: *homo sapiens*. Dm: *Drosophila melanogaster*.

In addition to transcriptome analysis of conserved signaling pathway components and regulatory genes, we also tried to identify genes known to be important for embryogenesis based on our transcriptome data. Here, we focused specifically on the *Pax* family genes. *Pax* genes, which are grouped into 4 subfamilies (*Pax1/9*, *Pax2/5/8*, *Pax3/7* and *Pax4/6*), encode a group of transcription factors that have been conserved through millions of years of evolution and play roles in early development [39–41]. According to the homology within the highly conserved paired domain, the putative horseshoe crab *Pax* genes were identified. A complete set of *Pax* family gene homologs was found in *T*. *tridentatus*. Phylogenetic tree was constructed by aligning the conserved paired domain with neighbor joining method using *Pseudomonas transposase* as outgroup (Fig 4). Genes belonging to *Pax4/6* subfamily branched out at the base of the tree. Genes from *Pax4/6* subfamily were divided into two clades. One included *Pax4/6* genes from *Homo sapiens* (*HsPax4* and *HsPax6*), *D*. *melanogaster* (*Dmtoy* and *Dmey*) and *T*. *tridentatus* (*TtPax4/6a* and *TtPax4/6b*). The second clade was composed of two sister groups with one containing the genes *Dmeyg* and *Dmtoe* from *D*. *melanogaster*, and the other consisting of *TtPax4/6c* and *TtPaX4/6d* from *T*. *tridentatus*. Interestingly, all the *Dmeyg*, *Dmtoe*, *TtPax4/6a* and *TtPaX4/6b* had a truncated paired domain. It is possible that the truncated paired domain already existed in the common ancestor of horseshoe crab and Drosophila. After split into two lineages, specific gene duplications occurred independently in horseshoe crab and Drosophila. The second clade includes *Pax2/5/8* subfamily genes. All members of *TtPax2/5/8* genes were gathered closely, indicating that relatively recent duplications appear to have occurred in horseshoe crabs. The third clade was composed of *Pax1/9* subfamily genes. *TtPax1/9a* branched out at the base against all other genes in this clade, whereas *TtPax1/9b* gathered with the vertebrate *Pax1* an*d Pax9* genes. The last clade was constituted of P*ax3/7* subfamily. The horseshoe crab *TtPax3/7a* was clustered with Drosophila *Dmgsb* and *Dmgsbn* genes.

While the majority of horseshoe crab genes of the *Pax* family show obvious homology to their respective subfamily, the placement of *TtPax3/7b* in the phylogenetic tree is ambiguous. It occupied the most basal position of the tree without obvious orthologs. The homeodomain sequence of *TtPax3/7b* was blasted against the GenBank database. Sequence comparison result showed that all the genes highly similar (over 78%) with *TtPax3/7b* homeodomain belonged to the Pax3/7 subfamily. Therefore, it is possible that *TtPax3/7b* genes belong to the Pax3/7 subfamily, but have been evolving at a rate that obscures its orthology.

**Fig 5:**
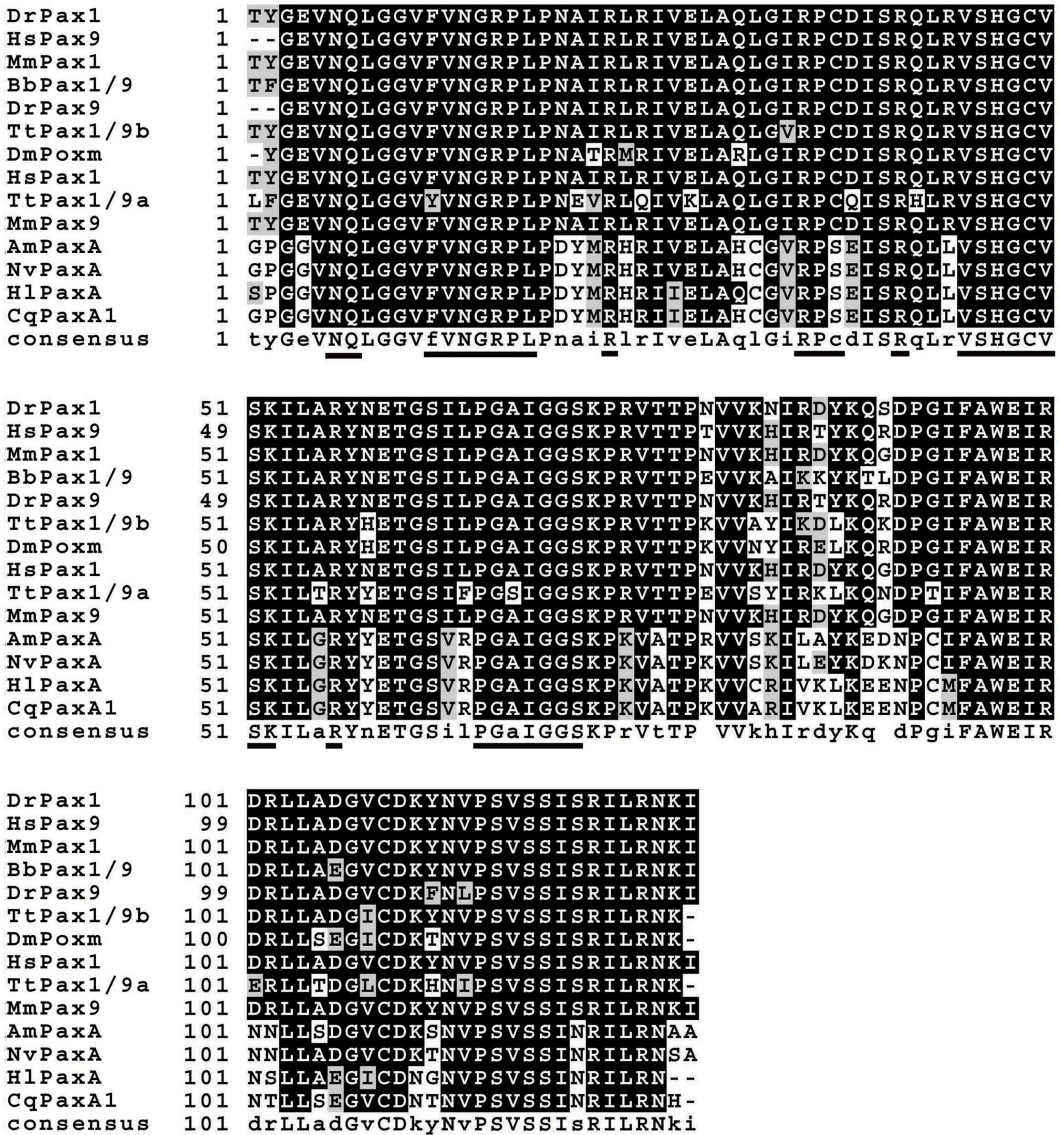
Multiple alignment of paired domain of Pax1/9 proteins from representative metazoan. The polypeptides were aligned using ClustalX 2.1 program and Boxshade online software (http://www.ch.embnet.org/software/BOX_form.html) were employed to highlight conserved amino acids. Conserved DNA binding motifs were underlined. The sequences used in the alignment are as follows: AmPaxA: *Acropora millepora* PaxA [GenBank: AAC15713.2], BbPax1/9: *Branchiostoma belcheri* Pax1/9 [GenBank: ABK54274.1], CqPaxA1: *Chrysaora quinquecirrha* PaxA1 [GenBank: AAB58292.1], DmPoxm: *Drosophila melanogaster* Poxm [GenBank: ABE69189.1], HlPaxA: *Hydra littoralis* PaxA [GenBank: AAB58290.1], DrPax1: *Danio rerio* Pax1 [GenBank: NP_001074061.1], DrPax9: *Danio rerio* Pax9 [GenBank: NP_571373.1], HsPax1: *Homo sapiens* Pax1 [GenBank: NP_006183.2], HsPax9: *Homo sapiens* Pax9 [GenBank: NP_006185.1], MmPax1: *Mus musculus* Pax1 [GenBank: AAK01146.1], MmPax9: *Mus musculus* Pax9 [GenBank: NP_035171.1] and NvPaxA: *Nematostella vectensis* PaxA [GenBank: AAW29066.1].

To further test the reliability of our database in gene discovery, the full length cDNAs encoding TtPax1/9a and TtPax1/9b proteins were cloned based on the partial sequences obtained from transcriptome assembly. The complete *TtPax1/9a* cDNA contained 258 bp of 5’ untranslated region (UTR), 681 bp of open reading frame (ORF) and 150 bp of 3’ UTR. The *TtPax1/9a* ORF can be translated into a polypeptide of 226 amino acids (aa) with a predicted molecular weight (MW) of 25.4 KDa. A conserved paired domain of 127 aa was included in the predicted protein sequence (S3 Fig). The full length *TtPax1/9b* cDNA contains 1893 bp, while the ORF is of 912 bp encoding 303 aa with a predicted MW of 33.2 KDa. A conserved paired domain with 127 aa in length is also present in the *TtPax1/9b* protein sequence (S4 Fig). A multiple alignment of the paired domain sequences of *Pax1/9* was performed in *T*. *tridentatus* and other representative metazoan species. As shown in Fig 5, the *Pax1* and *Pax9* showed high similarity among all the species examined. Interestingly, although both *T*. *tridentatus* and *D*. *melanogaster* belong to the Arthropoda, *TtPax1/9a* and *TtPax1/9b* showed higher protein identity to zebrafish (*Danio renio*), human (*Homo sapiens*) and mouse (*Mus musculus*) which belong to Chordata, comparing to *D*. *melanogaster*. Whereas, polypeptide identities of Pax paired domain between *T*. *tridentatus* and the Coelenterata species, including *Acropora millepora*, *Chrysaora quinquecirrha*, *Hydra littoralis* and *Nematostella vectensis*, were relatively low. In summary, our data demonstrated the immediate application of the transcriptome data for gene discovery in the “non-model” organism *T*. *tridentatus*.

### Tissue distribution of Pax1/9a and Pax1/9b

**Fig 6:**
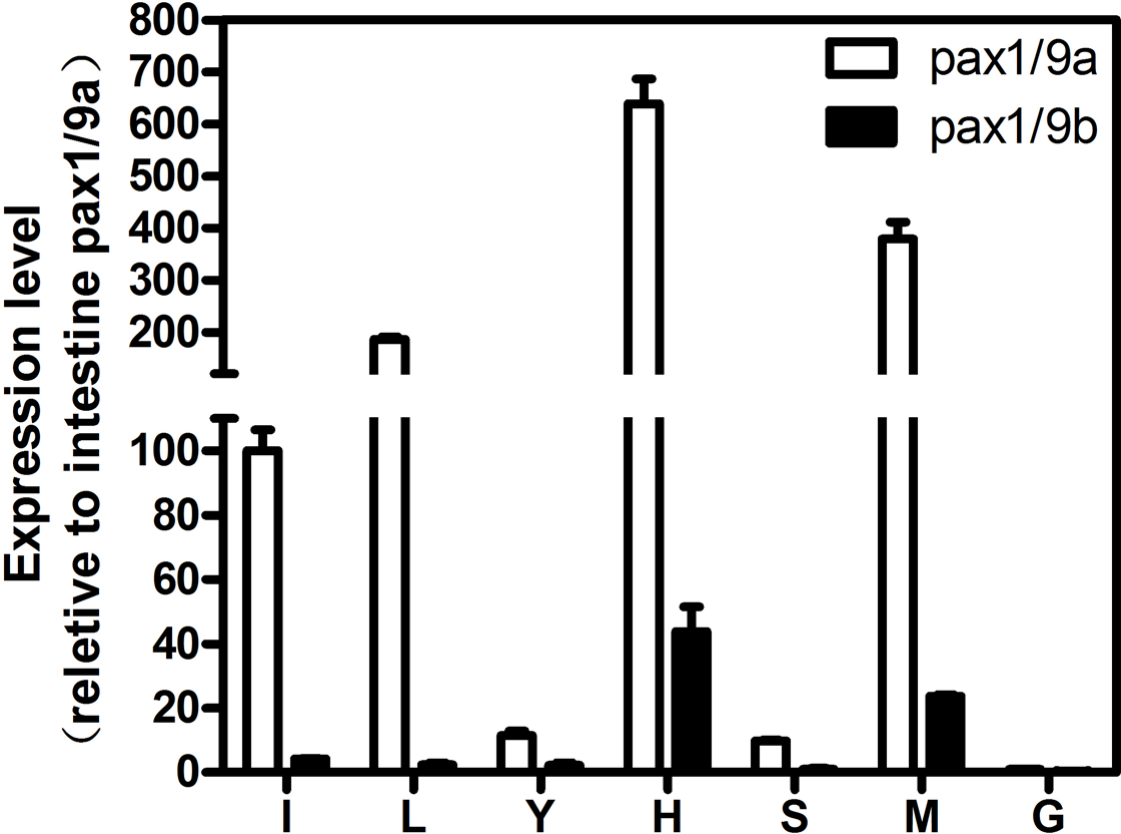
Tissue distribution of *TtPax1/9a* and *TtPax1/9b* determined by quantative real-time PCR. The *T*. *tridentatus GAPDH* transcript was used as internal standard. I, intestine; L: liver; Y, yellow connective tissue; H, heart; S: stomach; M, muscle; G, gill.

Expression patterns of *TtPax1/9a* and *TtPax1/9b* genes in different tissues of horseshoe crab were analyzed by quantitative RT-PCR. The highest expression level of *TtPax1/9a* was found in the heart (Fig 6). *TtPax1/9a* was also abundantly expressed in the muscle, liver and intestine. On the other hand, the expression level of *TtPax1/9a* in the stomach, yellow connective tissue and gill was significantly lower. Expression pattern of *TtPax1/9b* is similar to that of *TtPax1/9a*, albeit at much lower levels.

### *TtPax1/9b* partly rescues Poxm dysfunction-caused larval lethality of Drosophila

**Fig 7:**
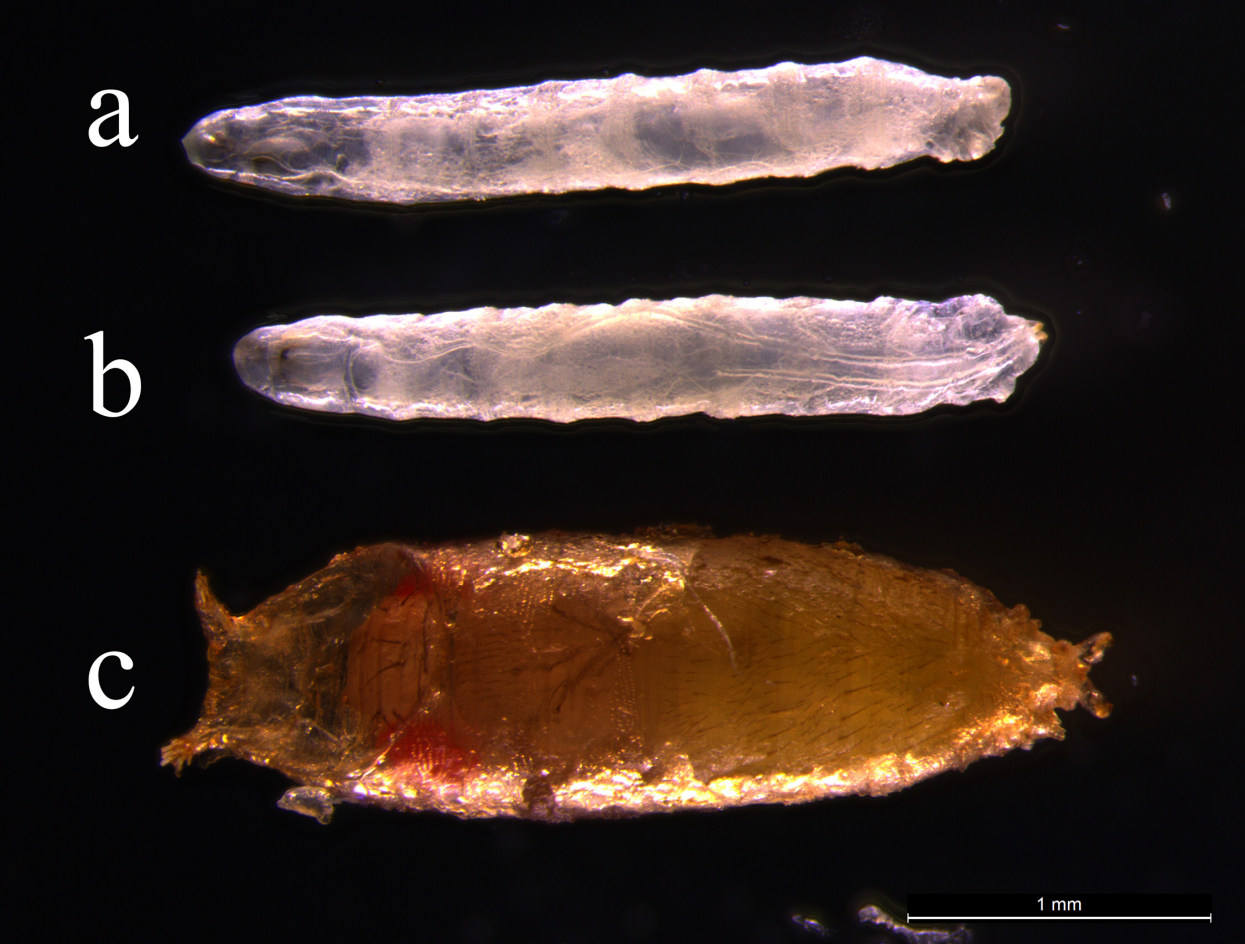
Rescue study of PoxmR361 mutant by the expression of *TtPax1/9a* and *TtPax1/9b* genes. (a) *Poxm*^*R361*^ mutant. (b) *Poxm*^*R361*^ mutant with *TtPax1/9a* gene expression. (c) *Poxm*^*R361*^ mutant with *TtPax1/9b* gene expression. Anterior is to the left.

Due to lack of genetic manipulation tools for the gene function study in *T*. *tridentatus*, we further explore the function of *TtPax1/9a* and *TtPax1/9b* genes by taking advantage of Drosophila model. Drosophila Pax gene *Pox meso* (*Poxm*) plays a crucial role in the early development. Our previous study showed that the loss-of-function mutant in *poxm*, *Poxm*^*R361*^, causes the Drosophila death [29]. According to our protein sequence alignment data, *TtPax1/9a* and *TtPax1/9b* genes are homologs of the Drosophila *poxm* gene. Therefore in this experiment, we investigated whether the *TtPax1/9a* and *TtPax1/9b* genes rescue *Poxm*^*R361*^-caused embryonic lethality of Drosophila. The *TtPax1/9a* and *TtPax1/9b* genes were expressed in the Drosophila under the control of the 8.4 kb *poxm* upstream region using GAL4/UAS system [29]. The *Poxm*^*R361*^ mutant alone (Fig 7A) and the *Poxm*^*R361*^ mutant with *TtPax1/9a* gene expression (Fig 7B) resulted in the Drosophila death at the larval stage. Surprisingly, The *Poxm*^*R361*^ mutant in the presence of *TtPax1/9b* survived at the pupal stage (Fig 7C). These results imply that the *TtPax1/9b* can at least partly rescue Poxm dysfunction-caused larval lethality of Drosophila.

## Conclusions

In this study, we, for the first time, performed *de novo* transcriptome sequencing of *T*. *tridentatus* embryos in the absence of a reference genome using Illumnia Solexa platform. Of 133,212 unigenes obtained, 33,796 were annotated by BLAST with NR, NT, Swiss-Prot, GO, COG and KEGG databases. We further identified a number of candidate genes potentially involved in embryonic development of *T*. *tridentatus*, shedding light on future study on the characterization and function of genes of interest and detailed molecular mechanisms underlying embryonic development of *T*. *tridentatus*. Moreover, the cloning and phylogenetic analysis of *Pax* family genes were performed, demonstrating that the transcriptome sequencing is a fast and reliable technology for high-throughput gene discovery in “non-model” organisms and for evolutionary developmental biology study as well. Rescue study further indicated that *TtPax1/9b* gene is functionally relative to Drosophila *Poxm* gene.

## Supporting Information

S1 Fig Sequence length distribution of transcripts and unigenes assembled from Illumina reads. All Illumina transcripts and unigenes with length over 200 bp were analyzed.
(TIF)

S2 Fig Contour plot of length and coverage distribution of annotated and unannotated unigenes. Transcripts were annotated using Blast2GO software. The Burrows-Wheeler Aligner (BWA) program was used for reads mapping. The color bar indicates log10 transformed count values.
(TIF)

S3 Fig Nucleotide and deduced amino acid sequence of *T*. *tridentatus Pax1/9a*. Numbers on the left indicate numbers of nucleotides or amino acids. Boxing indicates the conserved paired domain and * stands for putative stop codon. The polyadenylation signal is underlined.
(TIF)

S4 Fig Nucleotide and deduced amino acid sequence of *T*. *tridentatus Pax1/9b*. Numbers on the left indicate numbers of nucleotides or amino acids. Boxing indicates the conserved paired domain and * stands for putative stop codon.
(TIF)

S1 Table List of primer sequences used in this study. (DOCX)

S2 Table Paired domain amino acid sequences used in the phylogenetic analysis. Tt: *Tachypleus tridentatus*, Hs: *homo sapiens*, Dm: *Drosophila melanogaster*.
(DOCX)
