# Supplementary figures and images for: De Novo Assembly and Characterization of Early Embryonic Transcriptome of the Horseshoe Crab Tachypleus tridentatus
Source: PLoS One. 2016 Jan 5;11(1):e0145825. doi: 10.1371/journal.pone.0145825 (PMC4711587; doi:10.1371/journal.pone.0145825)

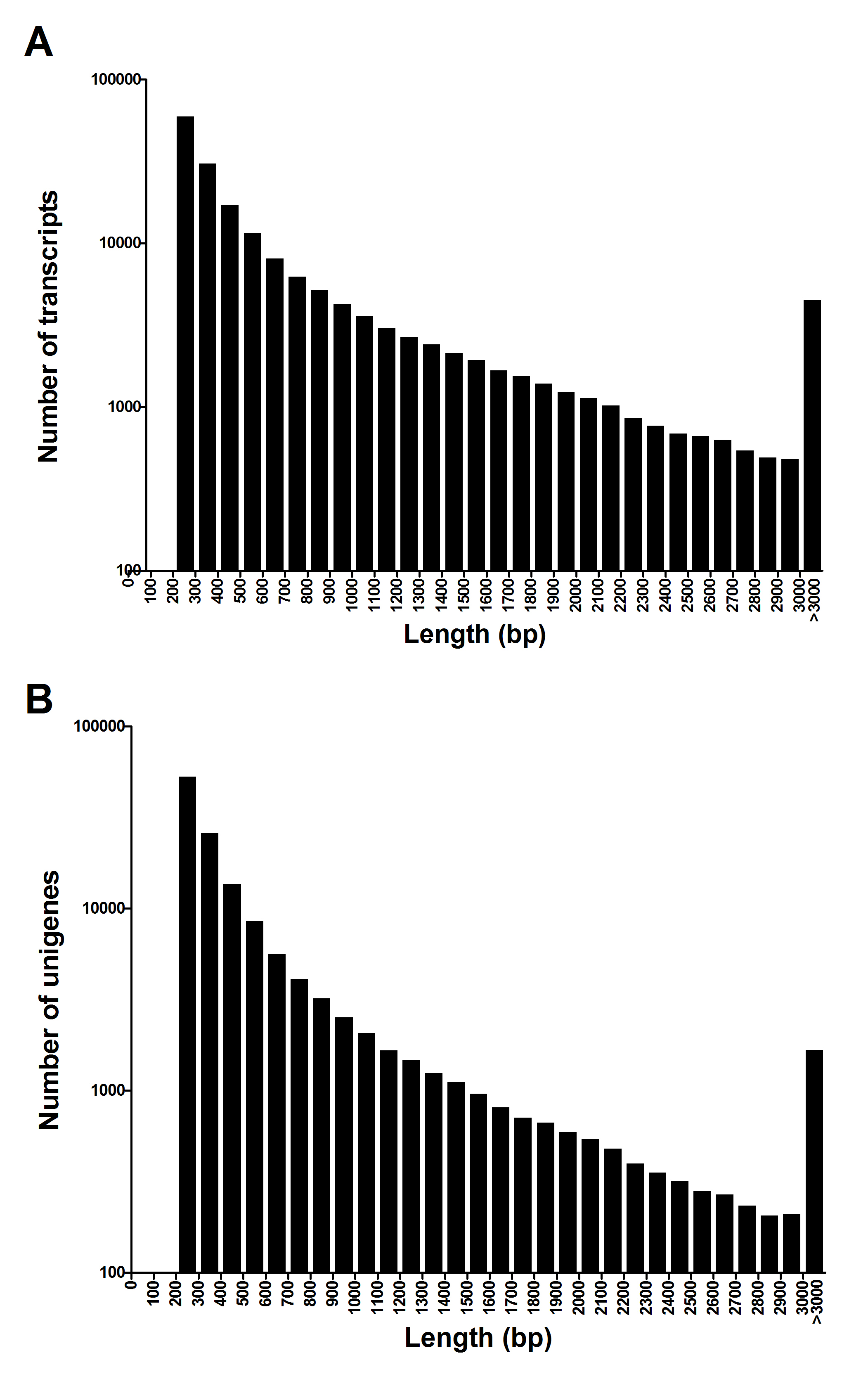

Supplement: S1 Fig — All Illumina transcripts and unigenes with length over 200 bp were analyzed. (TIF) [file pone.0145825.s001.tif]

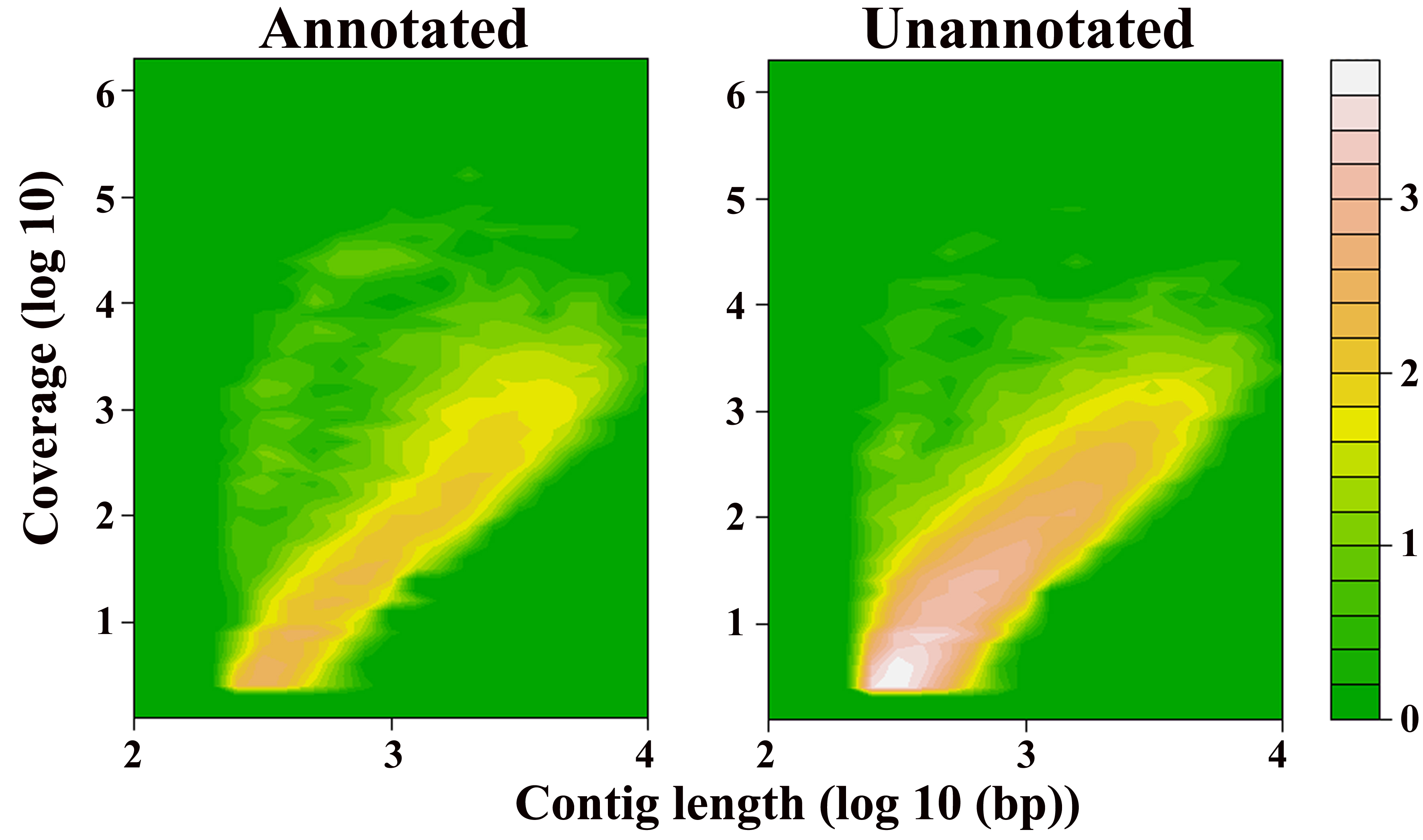

Supplement: S2 Fig — Transcripts were annotated using Blast2GO software. The Burrows-Wheeler Aligner (BWA) program was used for reads mapping. The color bar indicates log10 transformed count values. (TIF) [file pone.0145825.s002.tif]

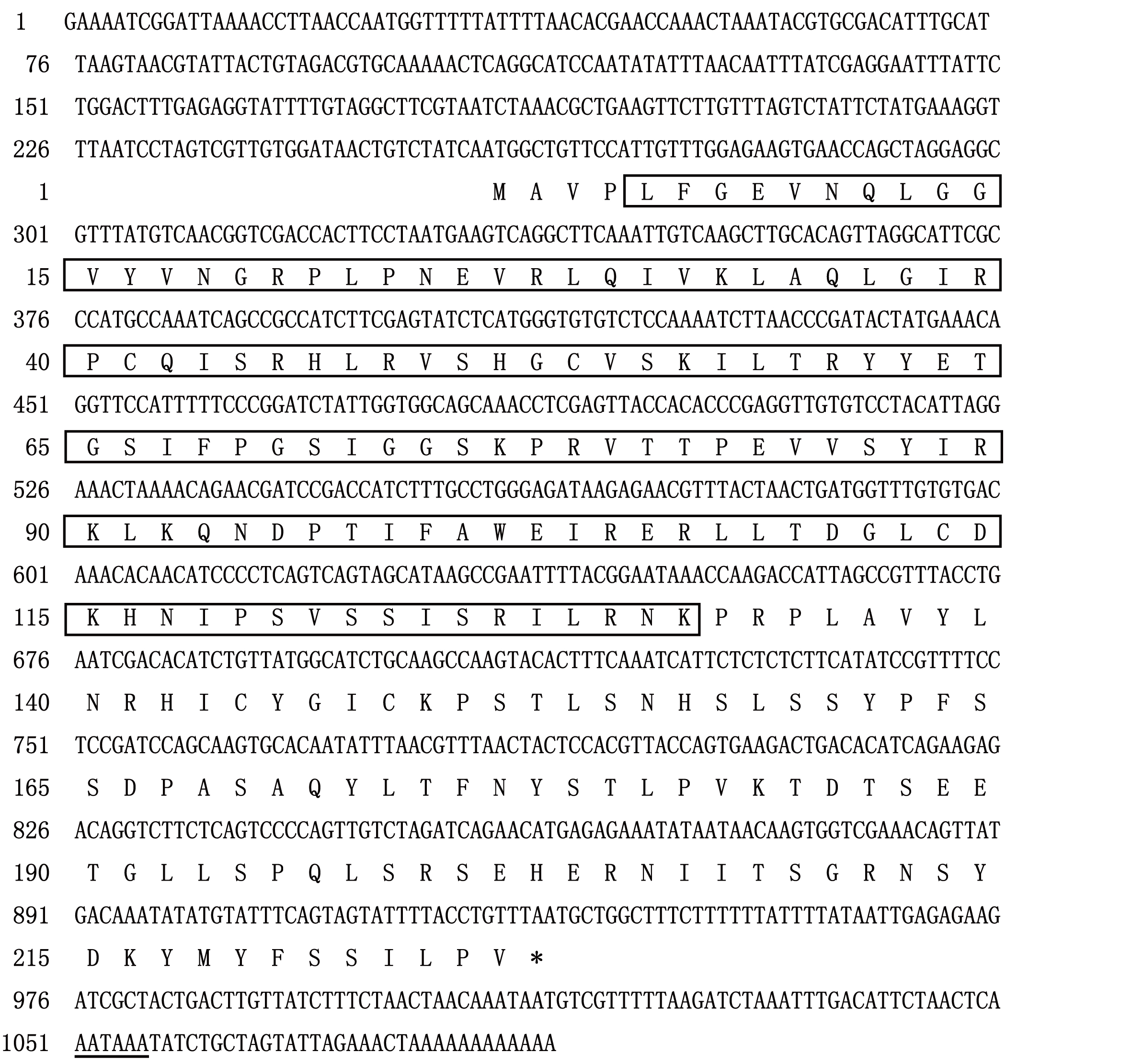

Supplement: S3 Fig — Numbers on the left indicate numbers of nucleotides or amino acids. Boxing indicates the conserved paired domain and * stands for putative stop codon. The polyadenylation signal is underlined. (TIF) [file pone.0145825.s003.tif]

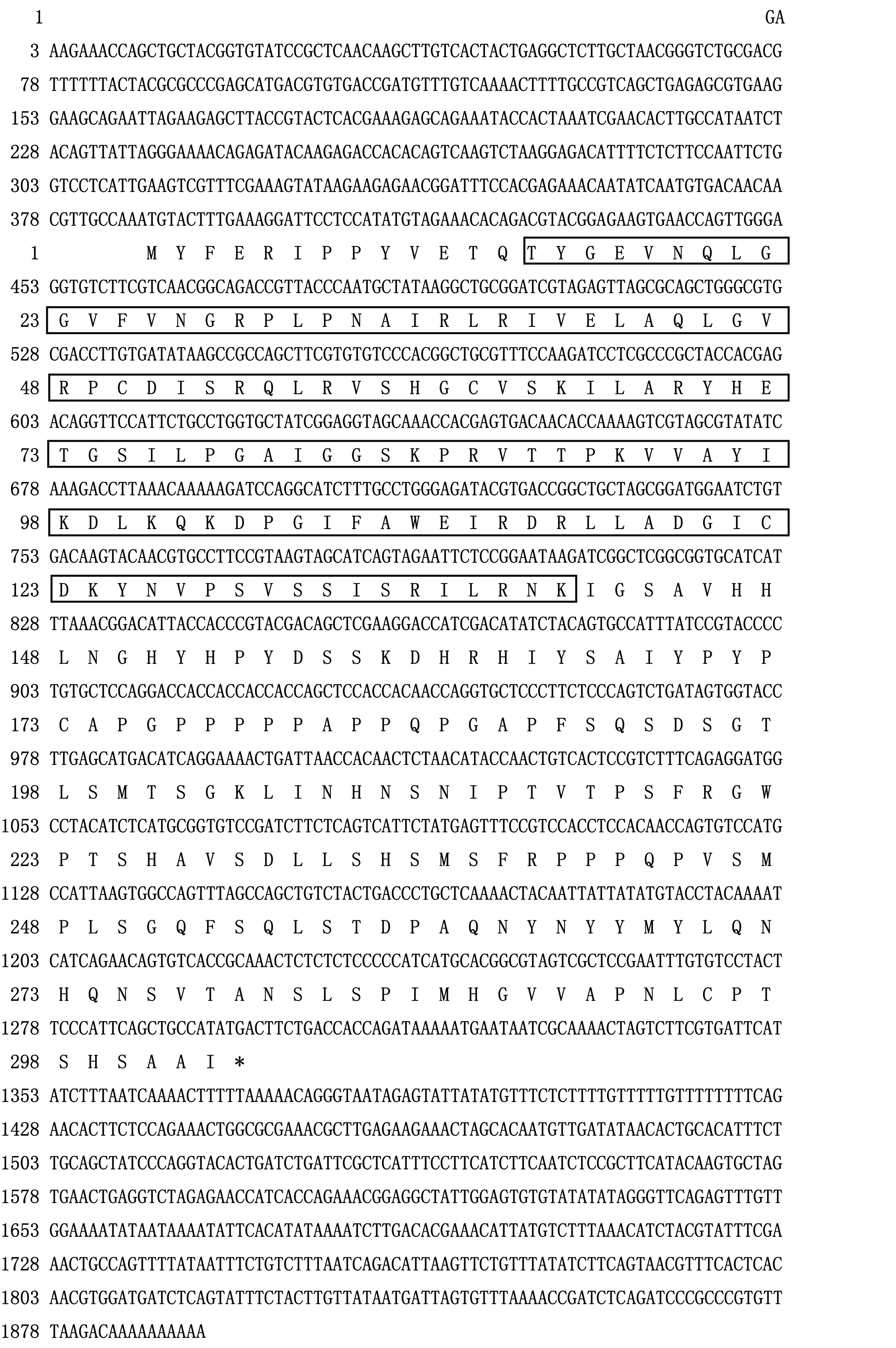

Supplement: S4 Fig — Numbers on the left indicate numbers of nucleotides or amino acids. Boxing indicates the conserved paired domain and * stands for putative stop codon. (TIF) [file pone.0145825.s004.tif]
