# Supplementary material for: De Novo Assembly and Characterization of Early Embryonic Transcriptome of the Horseshoe Crab Tachypleus tridentatus
Source: PLoS One. 2016 Jan 5;11(1):e0145825. doi: 10.1371/journal.pone.0145825 (PMC4711587; doi:10.1371/journal.pone.0145825)
Supplement: S1 Table — (DOCX) [file pone.0145825.s005.docx]

| **Primer name** | **Primer sequence (5’-3’)** | **Purpose** |
| --- | --- | --- |
| TtPax1/9a-F1 | AGACCATTAGCCGTTTACCTG | 3’-RACE |
| TtPax1/9a-F2 | CTTCTCAGTCCCCAGTTGTCT | 3’-RACE |
| TtPax1/9b-F1 | CATTCTATGAGTTTCCGTCCA | 3’-RACE |
| TtPax1/9b-F2 | CGCAAACTCTCTCTCCCCCAT | 3’-RACE |
| TtPax1/9a-R1 | CAACCTCGGGTGTGGTAACTCG | 5’-RACE |
| TtPax1/9a-R2 | GGTTCACTTCTCCAAACAATG | 5’-RACE |
| TtPax1/9b-R1 | CTGCCGTTGACGAAGACACCT | 5’-RACE |
| TtPax1/9b-R2 | CCTCCCAACTGGTTCACTTCT | 5’-RACE |
| 3Outer Primer | AAATCACTAGTGGAACGACGGTA | General primer for 3’-RACE |
| 3Inner Primer | CCTATAGTGAAATCACTAGTGGAGGATCCGCG | General primer for 3’-RACE |
| 5Outer Primer | CATGGCTACATGCTGACAGCCTA | General primer for 5’-RACE |
| 5Inner Primer | CGCGGATCCACAGCCTACTGATGATCAGTCGATG | General primer for 5’-RACE |
