# Supplementary material for: De Novo Assembly and Characterization of Early Embryonic Transcriptome of the Horseshoe Crab Tachypleus tridentatus
Source: PLoS One. 2016 Jan 5;11(1):e0145825. doi: 10.1371/journal.pone.0145825 (PMC4711587; doi:10.1371/journal.pone.0145825)
Supplement: S2 Table — Tt: Tachypleus tridentatus, Hs: homo sapiens, Dm: Drosophila melanogaster. (DOCX) [file pone.0145825.s006.docx]

| **Sequence name** | **Amino acid sequence** |
| --- | --- |
| HsPax1 | TYGEVNQLGGVFVNGRPLPNAIRLRIVELAQLGIRPCDISRQLRVSHGCVSKILARYNETGSILPGAIGGSKPRVTTPNVVKHIRDYKQGDPGIFAWEIRDRLLADGVCDKYNVPSVSSISRILRNKI |
| HsPax2 | GHGGVNQLGGVFVNGRPLPDVVRQRIVELAHQGVRPCDISRQLRVSHGCVSKILGRYYETGSIKPGVIGGSKPKVATPKVVDKIAEYKRQNPTMFAWEIRDRLLAEGICDNDTVPSVSSINRIIRTKV |
| HsPax3 | GQGRVNQLGGVFINGRPLPNHIRHKIVEMAHHGIRPCVISRQLRVSHGCVSKILCRYQETGSIRPGAIGGSKPKVTTPDVEKKIEEYKRENPGMFSWEIRDKLLKDAVCDRNTVPSVSSISRILRSKF |
| HsPax4 | MNQLGGLFVNGRPLPLDTRQQIVRLAVSGMRPCDISRILKVSNGCVSKILGRYYRTGVLEPKGIGGSKPRLATPPVVARIAQLKGECPALFAWEIQRQLCAEGLCTQDKTPSVSSINRVLRALQ |
| HsPax5 | GHGGVNQLGGVFVNGRPLPDVVRQRIVELAHQGVRPCDISRQLRVSHGCVSKILGRYYETGSIKPGVIGGSKPKVATPKVVEKIAEYKRQNPTMFAWEIRDRLLAERVCDNDTVPSVSSINRIIRTKV |
| HsPax6 | SHSGVNQLGGVFVNGRPLPDSTRQKIVELAHSGARPCDISRILQVSNGCVSKILGRYYETGSIRPRAIGGSKPRVATPEVVSKIAQYKRECPSIFAWEIRDRLLSEGVCTNDNIPSVSSINRVLRNLA |
| HsPax7 | GQGRVNQLGGVFINGRPLPNHIRHKIVEMAHHGIRPCVISRQLRVSHGCVSKILCRYQETGSIRPGAIGGSKPRVATPDVEKKIEEYKRENPGMFSWEIRDRLLKDGHCDRSTVPSVSSISRVLRIKF |
| HsPax8 | GHGGLNQLGGAFVNGRPLPEVVRQRIVDLAHQGVRPCDISRQLRVSHGCVSKILGRYYETGSIRPGVIGGSKPKVATPKVVEKIGDYKRQNPTMFAWEIRDRLLAEGVCDNDTVPSVSSINRIIRTKV |
| HsPax9 | AFGEVNQLGGVFVNGRPLPNAIRLRIVELAQLGIRPCDISRQLRVSHGCVSKILARYNETGSILPGAIGGSKPRVTTPTVVKHIRTYKQRDPGIFAWEIRDRLLADGVCDKYNVPSVSSISRILRNKI |
| DmPoxm | QYGEVNQLGGVFVNGRPLPNATRMRIVELARLGIRPCDISRQLRVSHGCVSKILARYHETGSILPGAIGGSKPRVTTPKVVNYIRELKQRDPGIFAWEIRDRLLSEGICDKTNVPSVSSISRILRNKL |
| Dmsv | GHGGVNQLGGVFVNGRPLPDVVRQRIVELAHNGVRPCDISRQLRVSHGCVSKILSRYYETGSFKAGVIGGSKPKVATPPVVDAIANYKRENPTMFAWEIRDRLLAEAICSQDNVPSVSSINRIVRNKA |
| DmPoxn | GQGRVNQLGGVFINGRPLPNHIRLKIVEMAASGVRPCVISRQLRVSHGCVSKILNRYQETGSIRPGVIGGSKPKVTSPEIETRIDELRKENPSIFSWEIREKLIKEGFADPPSTSSISRLLRGSD |
| Dmprd | GQGRVNQLGGVFINGRPLPNHIRHKIVEMAHHGIRPCVISRQLRVSHGCVSKILCRYQETGSIRPGAIGGSKPKVTTPDVEKKIEEYKRENPGMFSWEIRDKLLKDAVCDRNTVPSVSSISRILRSKF |
| Dmgsb | GQGRVNQLGGVFINGRPLPNHIRRQIVEMAAAGVRPCVISRQLRVSHGCVSKILNRFQETGSIRPGVIGGSKPRVATPDIESRIEELKQSQPGIFSWEIRAKLIEAGVCDKQNAPSVSSISRLLRGSS |
| Dmgsbn | GQGRVNQLGGVFINGRPLPNHIRLKIVEMAASGVRPCVISRQLRVSHGCVSKILNRYQETGSIRPGVIGGSKPKVTSPEIETRIDELRKENPSIFSWEIREKLIKEGFADPPSTSSISRLLRGSD |
| Dmey | CHSGVNQLGGVFVGGRPLPDSTRQKIVELAHSGARPCDISRILQVSNGCVSKILGRYYETGSIRPRAIGGSKPRVATAEVVSKISQYKRECPSIFAWEIRDRLLQENVCTNDNIPSVSSINRVLRNLA |
| Dmtoy | GHSGINQLGGVYVNGRPLPDSTRQKIVELAHSGARPCDISRILQVSNGCVSKILGRYYETGSIKPRAIGGSKPRVATTPVVQKIADYKRECPSIFAWEIRDRLLSEQVCNSDNIPSVSSINRVLRNLA |
| Dmeyg | EFSRFGLRGYDIAQHMLTQQGAVSKLLGSLRPGLIGGSKPKVATPTVVSKIEQYKRENPTIFAWEIRERLISEGVCTNATAPSVSSINRILRNRA |
| Dmtoe | ELSRFGLRGYDLAQHMLSQQGAVSKLLGTLRPGLIGGSKPKVATPTVVSKIEQYKRENPTIFAWEIRERLITEGVCTNATAPSVSSINRILRNRA |
| TtPax1/9a | LFGEVNQLGGVYVNGRPLPNEVRLQIVKLAQLGIRPCQISRHLRVSHGCVSKILTRYYETGSIFPGSIGGSKPRVTTPEVVSYIRKLKQNDPTIFAWEIRERLLTDGLCDKHNIPSVSSISRILRNKP |
| TtPax1/9b | TYGEVNQLGGVFVNGRPLPNAIRLRIVELAQLGVRPCDISRQLRVSHGCVSKILARYHETGSILPGAIGGSKPRVTTPKVVAYIKDLKQKDPGIFAWEIRDRLLADGICDKYNVPSVSSISRILRNKI |
| TtPax2/5/8a | HGCVSKILGRYYETGSIKPGVIGGSKPKVATPKVVEAISTYKKQNPTMFAWEIRDRLLADGVCDQDNIPSVSSINRIVRNKA |
| TtPax2/5/8b | SHGGVNQLGGMFVNGRPLPDMVRQRIVEMAHQGIRPCDISRQLRVSHGCVSKILGRFYETGSIKPGVIGGSKPKVATPKVVEAISLYKKQNPTMFAWEIRDRLLSDGVCDQESIPSVSSINRIVRNKA |
| TtPax2/5/8c | HGGINQLGGMFVNGRPLPDMVRQRIVEMAHQGVRPCDISRQLRVSHGCVSKILGRFYETGSIKPGVIGGSKPKVATPKVVDAISNYKKQNPTMFAWEIRDRLLADGICDQDNIPSVSSINRIVRNKA |
| TtPax2/5/8d | HGGVNQLGGVFVNGRPLPDIVRQRIVELAHQGVRPCDISRQLRVSHGCVSKILGRYYETGSIKPGVIGGSKPKVATPKVVDAIASYKKQNPTMFAWEIRDRLLADGICDQDNIPSVSSINRIVRNKA |
| TtPax3/7a | GQGRVNQLGGVFINGRPLPNHIRLKIVEMAAAGIRPCVISRQLRVSHGCVSKILNRYQETGSIRPGVIGGSKPRVASPDVEKKIEEYKKENPGIFSWEIRDRLVKEGYCDRGTAPSVSSISRLLRGNG |
| TtPax3/7b | RPCVISRKLKVSHGCVSKILNRYQETGSIRPGVKAESKPRATSPDEGQNIEDSQNKKACIYTWEMCDRLIKDGDCDKRSAITASSVSRTLRGGI |
| TtPax4/6a | TRQKIVELAHSGARPCDIARILQVSNGCVSKILGRYYETGSIRPRAIGGSKPRVATPPVVAKIAHYKRECPSIFAR |
| TtPax4/6b | INQLGGIYVNGRPLPDSTRQKIVELAHSGARPCDISRILQVSNGCVSKILGRYYETGSIRPRAIGGSKPRVATPDVVSNIAQYKRECPSIFAWEIRDRLLSDGICTNDNVPSVSSINRVLRNLA |
| TtPax4/6c | EFSPYGLRPYDFARHFLTSQTTVSKILGRYYETGSLRPGVIGGSKPKVATPTVVAKIEQYKRENPTIFAWEIRERLISERVCTNNTAPSVSSINRILRNRA |
| TtPax4/6d | EMTPYSLQPYDFVRHLLMSQNTVSKILGRCYETGALRQGIVGGSKPKVATPEVVAKIEQYKRENPTIFAWEIRERLISEGVCVNNTAPSVSSINRILRNRA |
| PsTrans | RHGTTSLFAALDVATGEVIGRLKRQHRSVEFLSFLPADVPIHLIMDNYATHKNDKVKAWLAAHPRYSIHFTPTSASWMNLVERFFSTLSEKWIKRQAHVSVKDLEASIEYYLETYNQNPKPFRWHK |
